# Quantitative Assessment of Antibody Internalization with Novel Monoclonal Antibodies against Alexa Fluorophores

**DOI:** 10.1371/journal.pone.0124708

**Published:** 2015-04-20

**Authors:** Sindy Liao-Chan, Barbara Daine-Matsuoka, Nathan Heald, Tiffany Wong, Tracey Lin, Allen G. Cai, Michelle Lai, Joseph A. D’Alessio, Jan-Willem Theunissen

**Affiliations:** Department of Discovery Research, Igenica Biotherapeutics, Burlingame, California, United States of America; CNR, ITALY

## Abstract

Antibodies against cell surface antigens may be internalized through their specific interactions with these proteins and in some cases may induce or perturb antigen internalization. The anti-cancer efficacy of antibody-drug conjugates is thought to rely on their uptake by cancer cells expressing the surface antigen. Numerous techniques, including microscopy and flow cytometry, have been used to identify antibodies with desired cellular uptake rates. To enable quantitative measurements of internalization of labeled antibodies, an assay based on internalized and quenched fluorescence was developed. For this approach, we generated novel anti-Alexa Fluor monoclonal antibodies (mAbs) that effectively and specifically quench cell surface–bound Alexa Fluor 488 or Alexa Fluor 594 fluorescence. Utilizing Alexa Fluor–labeled mAbs against the EphA2 receptor tyrosine kinase, we showed that the anti-Alexa Fluor reagents could be used to monitor internalization quantitatively over time. The anti-Alexa Fluor mAbs were also validated in a proof of concept dual-label internalization assay with simultaneous exposure of cells to two different mAbs. Importantly, the unique anti-Alexa Fluor mAbs described here may also enable other single- and dual-label experiments, including label detection and signal enhancement in macromolecules, trafficking of proteins and microorganisms, and cell migration and morphology.

## Introduction

The efficacy of certain antibody-based therapies, such as antibody-drug conjugates (ADCs), depends not only on binding affinity and specificity to the antigen, but also on internalization [1–3]. Antibodies or antibody fragments can deliver various payloads, for example small molecules and proteins, to target cells that express the surface antigen. Rapid endocytosis of antigen-antibody complexes and subsequent release of the payload into the cytosol enables specific delivery to cells that overexpress the antigen and prevents systemic exposure.

Different techniques have been developed to identify antibodies or antibody fragments that undergo rapid internalization. Cellular uptake can be assessed with radioisotope-labeled antibodies by removing membrane-associated antibody with an acidic buffer [4, 5] or a protease [6]. However, these stripping procedures can have deleterious effects on cells [7]. Fluorescent dyes are also used to monitor antibody internalization. In a flow cytometry–based assay, a fluorescently labeled secondary antibody may be used to measure how much antibody remains surface-bound after an incubation period. Alternatively, cells may be treated with fluorescently labeled primary antibodies prior to cell surface fluorescence quenching with an anti-fluorophore antibody [7, 8]. An important advantage of using an anti-fluorophore antibody is that a gain of signal on the flow cytometer, rather than a loss of signal, is a measure of antibody internalization. Various processes, including internalization or fast antibody off-rates, can trigger a loss of signal. While internalization can also be monitored on a flow cytometer with pH sensitive dyes, which are non-fluorescent at neutral pH and exhibit increasing fluorescence as the pH becomes more acidic in the endocytic compartment, the relatively small increase in fluorescence over the cellular pH range in question limits their applicability [9, 10]. Immunofluorescence microscopy–based colocalization with endosomal proteins is also employed to monitor cellular uptake [7, 11–13]. Unlike immunofluorescence microscopy, flow cytometry with an anti-fluorophore antibody enables a more rapid and quantitative assessment of antibody internalization, and potentially greater throughput.

In this report we generated anti-Alexa Fluor antibodies against two Alexa fluorophores for which mAbs have not been previously reported. The existing polyclonal antibody against Alexa Fluor 488 only enables single-label experiments, cross-reacts with the carrier protein used for immunization, and cannot be produced recombinantly [7]. The anti-Alexa Fluor mAbs described here quenched fluorescence and were validated in single-label and dual-label flow cytometry–based internalization assays utilizing a pair of non-competing antibodies against a cell surface antigen. Other types of single-label and dual-label experiments may also benefit from these novel anti-Alexa Fluor reagents.

## Material and Methods

### Anti-Alexa Fluor mAb generation

The anti-Alexa Fluor mAbs were derived by immunizing mice (129S6/SvEvTac and SJL/J) with keyhole limpet hemocyanin protein (KLH) conjugated to A488 or A594 using a 13-day rapid immunization multiple sites protocol [14]. The mouse studies described herein were approved by Igenica Biotherapeutics’ Institutional Animal Care and Use Committee (IACUC Protocol IGA.001 issued to Jan-Willem Theunissen). Mice were euthanized in a carbon dioxide chamber. Target-specific hybridomas were established by electrofusion of lymphocytes isolated from peripheral lymph nodes with the myeloma cell line Sp2/mIL-6 (ATCC, Manassas, VA). The non-quenching chimeric IgG1 isotype control was derived from C1.18.4 (ATCC), a myeloma line that expresses a mouse IgG2a of unknown specificity, by sequencing the rearranged variable regions (S1 Table). The germline identity and the complementarity determining regions (CDRs) of the sequenced variable regions of the quenching mAbs were identified with IMGT (the international ImMunoGeneTics information system http://www.imgt.org) and Kabat’s definition, respectively. To produce the quenching mAbs, the chimeric IgG1 isotype control and the anti-EphA2 mAb 1C1 [15], the sequenced VH regions (including the signal sequences) were cloned in frame with the constant region of the human IgG1 heavy chain (pFUSE-CHIg-hG1, Invivogen, San Diego, CA) using the restriction enzymes EcoRI and NheI. The sequenced VK regions (including the signal sequences) were cloned in frame with the human kappa light chain (pFUSE-CLIg-hK, Invivogen) using the restriction enzymes AgeI and BsiWI. The mAbs were expressed at a 1:2 ratio of heavy to light chain plasmid in the Expi293 Expression System (Life Technologies, Carlsbad, CA), and purified with a standard MabSelect SuRe purification protocol (GE Healthcare, Piscataway, NJ).

### Antibody reagents and cell lines

Cell lines (PC-3, HEC-1-A) were obtained from ATCC and cultured as suggested. Antibodies were obtained from the following sources: mouse anti-human EphA2 clone 371805 (MAB3035 [abbreviated as 3035], R&D Biosystems, Minneapolis, MN); Alexa Fluor 488 goat anti-mouse or anti-human IgG antibody and Alexa Fluor 546 goat anti-mouse or anti-rabbit IgG antibody (Life Technologies); anti-LAMP1 clone D2D11, (Cell Signaling, Danvers, MA). Alexa Fluor 647–Phalloidin (cat. no. 8940, Cell Signaling) was used to stain the actin cytoskeleton. Alexa Fluor antibody conjugates were generated using Alexa Fluor 488 and Alexa Fluor 594 5-sulfodichlorophenol esters (Life Technologies). Excess Alexa Fluor dye was removed from the antibody dye conjugate preparation by gel filtration (Thermo Pierce, Rockland, IL). The degree of Alexa Fluor labeling was determined by conducting SDS-PAGE on 1.5 microgram of each dye-conjugated antibody, and scanning unstained or Coomassie-stained gels on a Typhoon (GE Healthcare) or a conventional scanner, respectively. We used a blue laser (488 nm) with a 526 SP emission filter for detection of A488 and a green laser (532 nm) with a 610 BP emission filter for detection of A594. The relative quantities of Alexa Fluor dye on the heavy and light chains were established using ImageQuantTL (GE Healthcare), while the relative quantities of naked antibody were measured using ImageJ [16].

### Antibody binding studies

Kinetic measurements for the anti-EphA2 mAbs were established with an Octet QK384 (ForteBio, Menlo Park, CA) using anti-human IgG Fc biosensors (ForteBio, Part No. 18–5064) for 1C1 and anti-mouse IgG Fc biosensors (ForteBio, Part No. 18–5090) for 3035. Anti-EphA2 mAbs were immobilized onto the biosensor surface for 2.5 min and a baseline step was recorded by moving the sensors into 1X Kinetics Buffer (ForteBio, Part No. 18–5032) for 2 min. Association of an eight-point two-fold titration of the recombinant extracellular domain of EphA2 (R&D Systems, Cat. No. 3035-A2-100) starting at 300 nM for 1C1 mAbs and at 200 nM for 3035 mAbs was measured for 5 min and dissociation was recorded by moving the biosensors to 1X Kinetics Buffer for 20 min. Reference biosensors were dipped in 1X Kinetics Buffer during the association step. The sample plate was agitated at 1000 rpm throughout the entire experiment. Data was aligned to the baseline step, reference biosensor subtracted, inter-step corrected by alignment to the dissociation step, and filtered using the Savitzky-Golay equation in Octet Data Analysis 7.0 software. Kinetic data was analyzed and fitted globally using a 1:1 Langmuir binding model. The K_D_ was calculated by dividing the k_dis_ by the k_on_.

Cell-based antibody binding studies were conducted as described [17]. 1x10^5^ cells were collected with Cellstripper (Mediatech, Manassas, VA), then incubated with an eleven-point 1:2 dilution series of anti-human EphA2 antibody dye conjugate starting at 200 nM for 3 hr at 4°C. After a wash step and addition of the viability dye 4',6-diamidino-2-phenylindole (DAPI), 10,000 events were collected on a MACS-Quant flow cytometer (Miltenyi, Auburn, CA). The median fluorescence intensities (MFIs) at each dilution were plotted and an apparent Kd was derived by using the one-site specific binding with Hill slope equation (Prism, GraphPad Software Inc, La Jolla, CA). The Kd values and their associated 95% confidence intervals are reported in S1 Fig.

### Quenching activity of anti-Alexa Fluor antibodies

**Fig 1:**
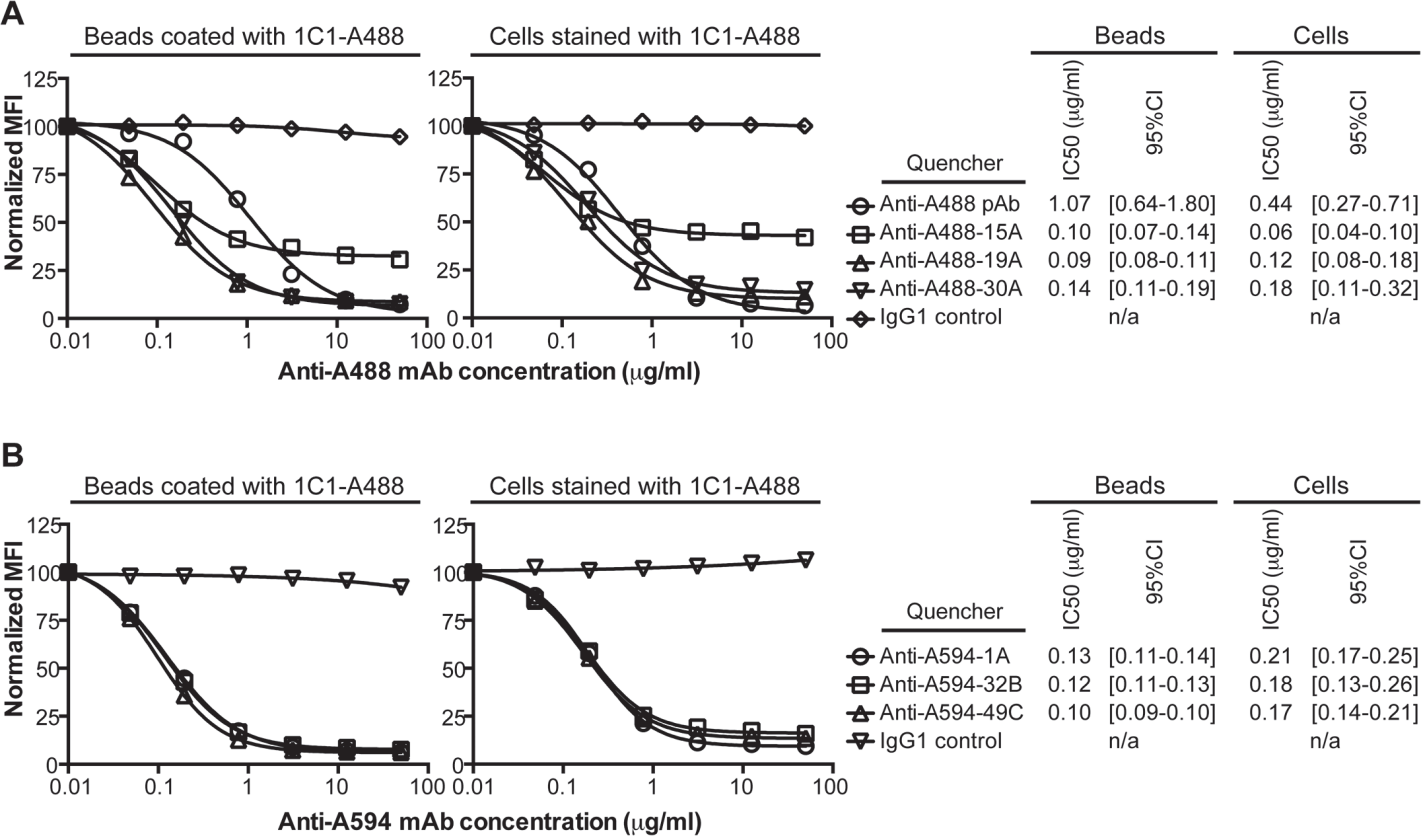
Quenching by anti-Alexa Fluor mAbs. (**A**) Fluorescence of Alexa Fluor 488 (A488) on microbeads coated with 1C1-A488 or PC-3 cells stained with 1C1-A488 was quenched with a titration of the benchmark, a rabbit anti-A488 polyclonal, or 1 of 3 anti-A488 mAbs. One representative experiment of multiple is shown. (**B**) Fluorescence of Alexa Fluor 594 (A594) on microbeads coated with 1C1-A594 or PC-3 cells stained with 1C1-A594 was quenched with a titration of 1 of 3 anti-A594 mAbs. One representative experiment of multiple is shown. (**A, B**) Median fluorescence intensities (MFIs) at each anti-A488 or anti-A594 mAb concentration were normalized against a buffer control. The chimeric IgG1 isotype control was used as a non-quenching mAb control. The IC50 values (microgram/ml) of quenching and the corresponding 95% confidence intervals (95% CI) are listed for both the microbead- and cell-based titrations.

PC-3 cells or anti-human IgG microbeads (Miltenyi) were labeled on ice with 100 nM of anti-human EphA2 antibody, a concentration at which staining is saturated (S1 Fig). After a wash step, the cells or beads were incubated for 30 min with buffer or a 6-point 1:4 dilution series of anti-Alexa Fluor mAb starting at 50 microgram/ml. At 10 microgram/ml of anti-Alexa Fluor mAb or a higher concentration, quenching is saturated for 1C1 conjugates (Fig 1) and 3035 conjugates. After another wash step and addition of DAPI, 20,000 live cells or beads were acquired on a MACS-Quant VYB. The median fluorescence intensity (MFI) at each anti-Alexa Fluor mAb concentration was normalized against the buffer control to obtain a normalized MFI percentage. Half maximal inhibitory concentration (IC50) values were derived by using the log(inhibitor) versus response equation in Prism. The IC50 values and their 95% confidence intervals are reported in Fig 1.

### Flow cytometry–based internalization assay using cell surface fluorescence quenching

Cell surface quenching experiments were conducted as described [7]. PC-3 cells were collected with Cellstripper, pre-incubated for 30 min on ice with 15 microgram/ml antibody conjugate in PC-3 culture medium, washed three times with PC-3 culture medium, and incubated at 37°C for the indicated time intervals. Cells were rapidly chilled and incubated for 30 min with (quenched samples) or without (unquenched samples) 50 microgram/ml of anti-A488 or anti-A594 antibody in PBS/0.1%BSA. In unquenched samples 50 microgram/ml of the chimeric IgG1 isotype control antibody was added. 1.5x10^4^ live events were acquired on a MACS-Quant VYB after staining the cells with DAPI. On the MACS-Quant VYB the violet laser was used for the excitation of DAPI, the blue laser for the excitation of A488, and the yellow laser for the excitation of A594. The median A488 or A594 fluorescence intensity for viable cells was established using FlowJo software (Tree Star, Ashland, OR). Internalized fluorescence was calculated from quenched and non-quenched sample data by correcting for incomplete surface quenching:

1 –(N_1—_Q_1_)/(N_1 –_(N_1_Q_0_/N_0_)) with N_1_ = Unquenched MFI at each time point (t_1_); Q_1_ = Quenched MFI at t_1_; Q_0_ = Quenched MFI for the sample kept on ice (t_0_); N_0_ = Unquenched MFI at t_0_. When cells were incubated with two Alexa Fluor antibody conjugates, each anti-Alexa Fluor antibody was added at 50 microgram/ml during the quenching step.

Internalized fluorescence was plotted and a curve fit was obtained in Prism by nonlinear regression with the one-phase association equation: Y = Y_0_ + (Plateau—Y_0_) * (1—exp(-K*x)) with Y_0_ = 0 when X (time) is zero and Plateau = 100. The summary statistic half-time is the time at which internalized fluorescence is equal to fifty percent and enabled comparison of internalization for the different dye-conjugated anti-EphA2 mAbs. Half-time is in the time units on the X-axis and is computed as the reciprocal of ln(2)/K. For each curve fit the 95% confidence interval for half-time is reported.

### Indirect immunofluorescence

PC-3 cells were seeded into six-well plates at ~30% confluence and were stained with 5 microgram/ml of EphA2 antibody (1C1 or 3035) for 30 min at 4°C. After 3 washes the cells were incubated at 37°C for the indicated intervals in growth medium. Cells were fixed in 3% paraformaldehyde (PFA) in phosphate buffered saline (PBS). After quenching the PFA with 0.1 M glycine in PBS, cells were permeabilized with a staining buffer (0.4% saponin, 1% BSA, 2% normal goat serum in PBS). The LAMP1 marker antibody incubation, the fluorophore-conjugated secondary antibody incubation, and wash steps were performed in the staining buffer. Cells were imaged at The Gladstone Histology and Light Microscopy Core with a Zeiss LSM510 confocal microscope (Zeiss, Oberkochen, Germany) equipped with a C-Apochromat 63X/1.2W CORR objective lens. The settings for the three different channels were as follows: Cy5 14% of 633 nm laser, 132 micron pinhole, BP650-710 IR filter; TRITC 100% of 543 nm laser, 126 micron pinhole, BP565-615 IR filter; GFP 17% of 488 nm laser, 128 micron pinhole, BP500-530 IR filter. A control in which 1C1 or 3035 were each detected in two different channels showed colocalization of the secondary antibody reagents (S2 Fig), suggesting that these settings were appropriate for assessment of colocalization.

## Results

### Generation and characterization of anti-Alexa Fluor reagents

To develop an assay in which internalization of two different antibodies can be measured concurrently, we generated anti-Alexa Fluor antibodies against two Alexa Fluor dyes with different chemical structures, Alexa Fluor 488 (A488) and Alexa Fluor 594 (A594) [18]. Thirty-two anti-A488 and 60 anti-A594 antibody supernatants from hybridomas were assayed for their ability to quench fluorescence of A488 and A594 conjugated to antibodies captured by microbeads.

Upon antibody purification, quenching by three prioritized anti-A488 and three prioritized anti-A594 mAbs was confirmed on microbeads coated with an antibody-dye conjugate or EphA2-positive PC-3 cells stained with an antibody-dye conjugate (Fig 1). While the anti-A488 mAb 15A quenched 65% of A488 fluorescence, 19A and 30A quenched greater than 85% of A488 fluorescence (Fig 1A). The magnitude of A488 quenching by 19A and 30A was equivalent to the benchmark, an anti-A488 rabbit polyclonal antibody. However, the IC50 values of the anti-A488 mAbs were at least 2.5-fold lower than the value for the benchmark, likely due to the fact that a portion of the polyclonal preparation bound KLH (S3 Fig). The anti-A594 mAbs 1A, 32B and 49C quenched more than 85% of A594 fluorescence in both the microbead and the cell-based assays (Fig 1B)

The anti-A488 and anti-A594 mAbs did not bind or quench A594 and A488, respectively (S3 and S4 Figs).

**Fig 2:**
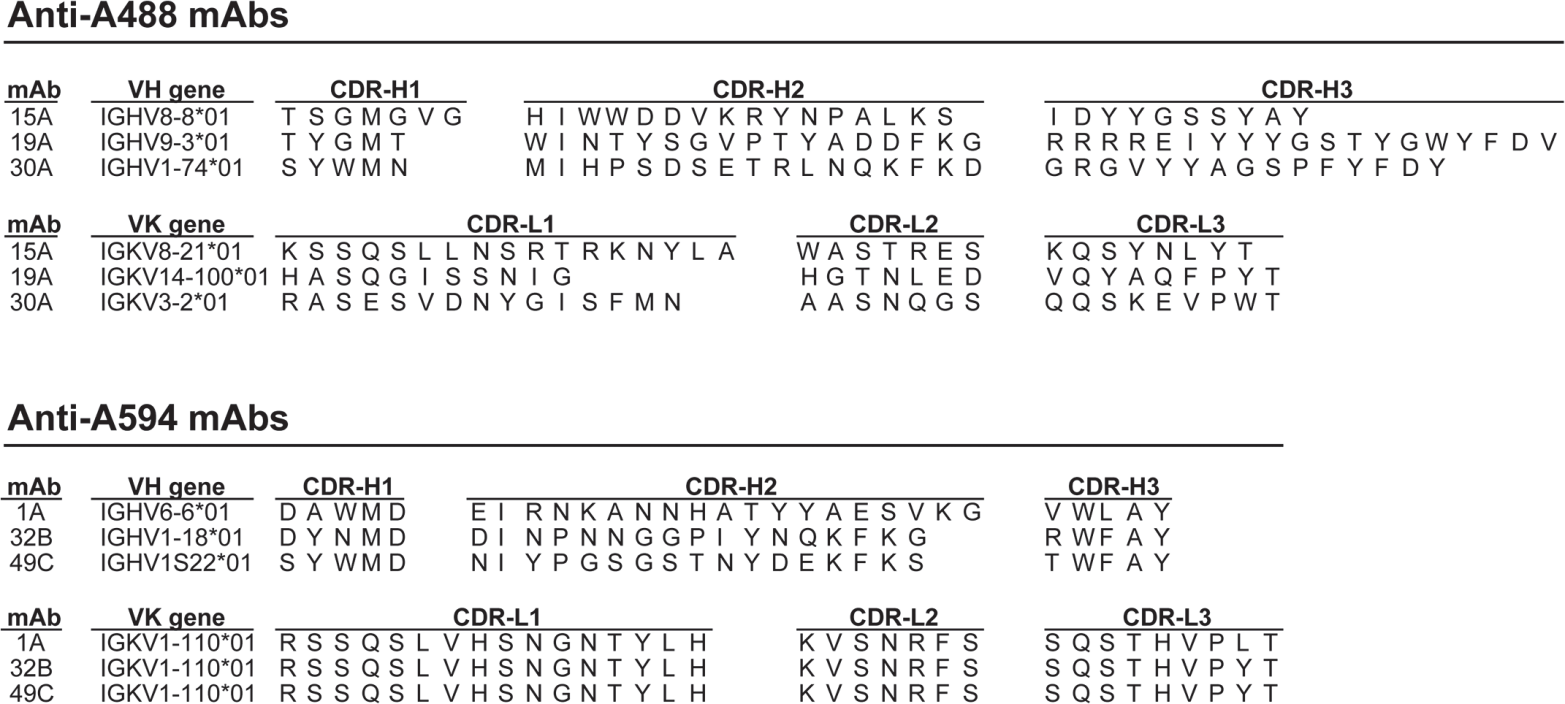
Variable region gene usage and CDRs for the anti-Alexa Fluor mAbs. The VH and VK genes and heavy and light chain complementarity determining regions (CDRs) for 3 anti-A488 and 3 anti-A594 mAbs are listed. The complete variable region sequences are presented in the S1 Table.

Antibody sequencing was conducted to evaluate anti-Alexa Fluor mAb diversity and to produce the mAbs recombinantly. None of the complementarity determining regions (CDRs) of the anti-A488 mAbs 15A, 19A and 30A shared more than 80% sequence identity (Fig 2 and S1 Table). Tyrosine, a residue previously implicated in fluorescence quenching [19], was present four to five times in the third CDR of the heavy chain of all anti-A488 mAbs. The anti-A594 mAbs shared at least 80% sequence identity in CDR-H3 and all light chain CDRs (Fig 2 and S1 Table).

### Identification of two non-competing mAbs against a cell surface antigen

We screened antibodies against various targets to identify two non-competitive mAbs with different internalization rates. A difference in cellular uptake between two mAbs enables us to gauge the ability of the internalization assay to discriminate fast from slow internalizers. Non-competitive binding of the two mAbs allows one to interrogate the interplay between two mAbs and their receptor.

**Fig 3:**
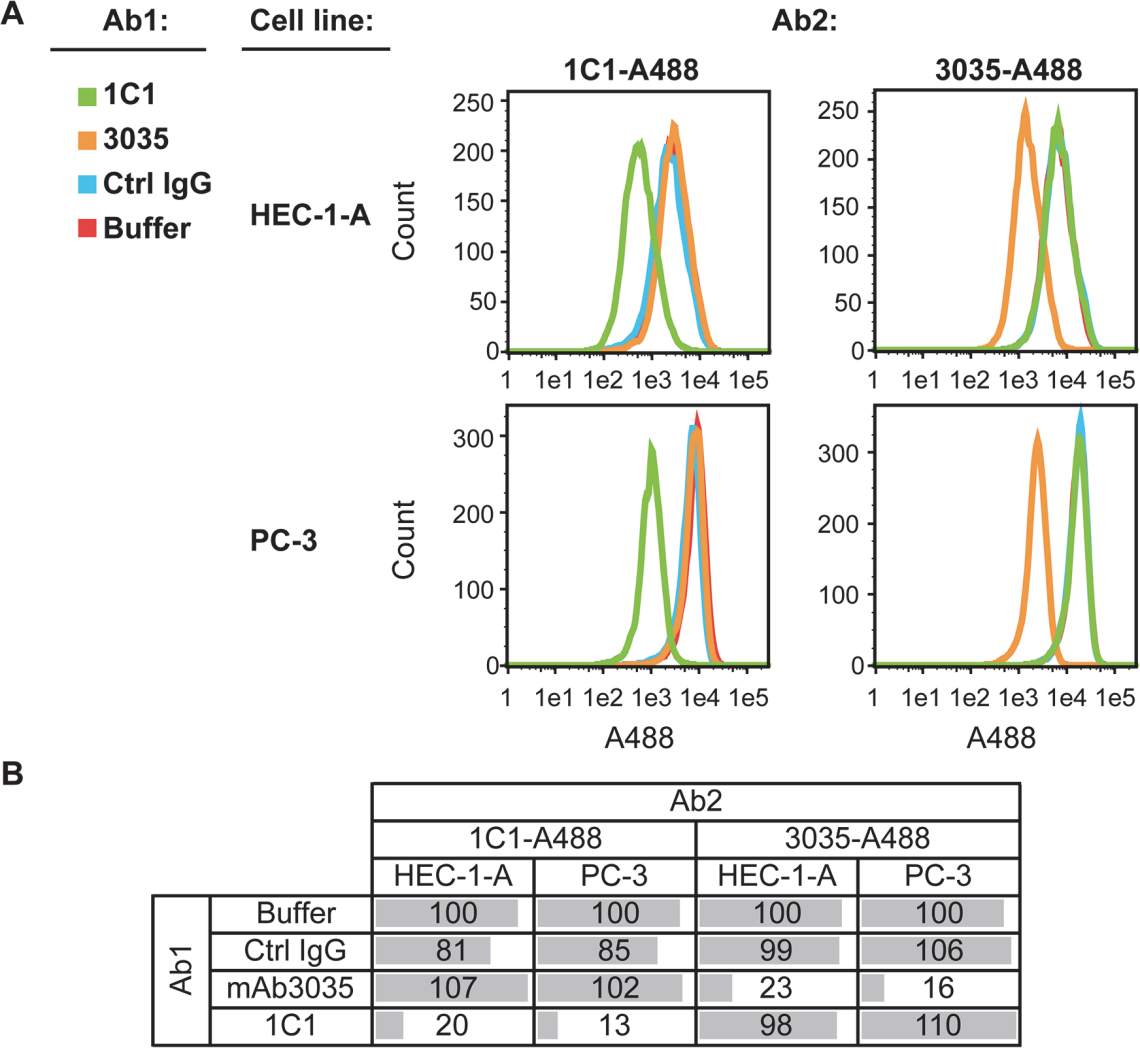
Identification of non-competing anti-EphA2 mAbs. (**A**) Histograms for EphA2-positive cell lines HEC-1-A and PC-3 first stained with unlabeled EphA2 mAb 1C1 or 3035, an IgG control, or buffer only, followed by staining with either labeled 1C1 (1C1-A488) or 3035 (3035-A488). A488 fluorescence is detected by conducting flow cytometry on 10,000 live, single cells. (**B**) Percent staining by 1C1-A488 or 3035-A488 relative to the HEC-1-A or PC-3 samples first incubated with buffer only.

The EphA2 receptor tyrosine kinase was one of the targets profiled. After cell binding, the mAb 1C1 induces tyrosine phosphorylation, fast internalization and degradation of the EphA2 receptor [15, 20]. In Fig 3, 1C1 and another anti-EphA2 mAb, 3035, did not compete for binding to EphA2 in two cancer cell lines. In PC-3 cells for example, pre-incubation with 3035 reduced binding of 3035-A488 by 84% relative to the no antibody control (Fig 3B). On the other hand, 3035 had no effect on binding of 1C1-A488, because 1C1 binding was equivalent in the absence or presence of 3035 (Fig 3B), indicating that these two mAbs have non-overlapping epitopes. Previously, it was shown that 1C1 binds an epitope distinct from that of two other anti-EphA2 mAbs [15].

### Anti-Alexa Fluor mAbs in quantitative flow cytometry–based internalization assay

To establish the utility of the anti-Alexa Fluor mAbs in the flow cytometry–based internalization assay described herein, we conjugated 1C1 and 3035 with either A488 or A594. To ensure that dye conjugation did not disrupt antigen binding, we evaluated binding of naked and dye-conjugated antibodies by measuring binding of immobilized antibody to recombinant EphA2 extracellular domain protein. While the K_D_ of naked and dye-conjugated 3035 was between 1.9 and 3.7 nM, the K_D_ of naked and dye-conjugated 1C1 was between 62 and 115 nM (S1 Fig), consistent with reported values [15]. We also evaluated apparent affinities of the antibody conjugates in a cell-based format: while the 1C1 conjugates had apparent affinities between 1.2 nM and 2.7 nM, the 3035 conjugates had apparent affinities of 0.6 nM (S1 Fig). The difference in protein-based affinity and cell-based apparent affinity measurements was likely due to avidity effects in the cell-based assay format [15].

**Fig 4:**
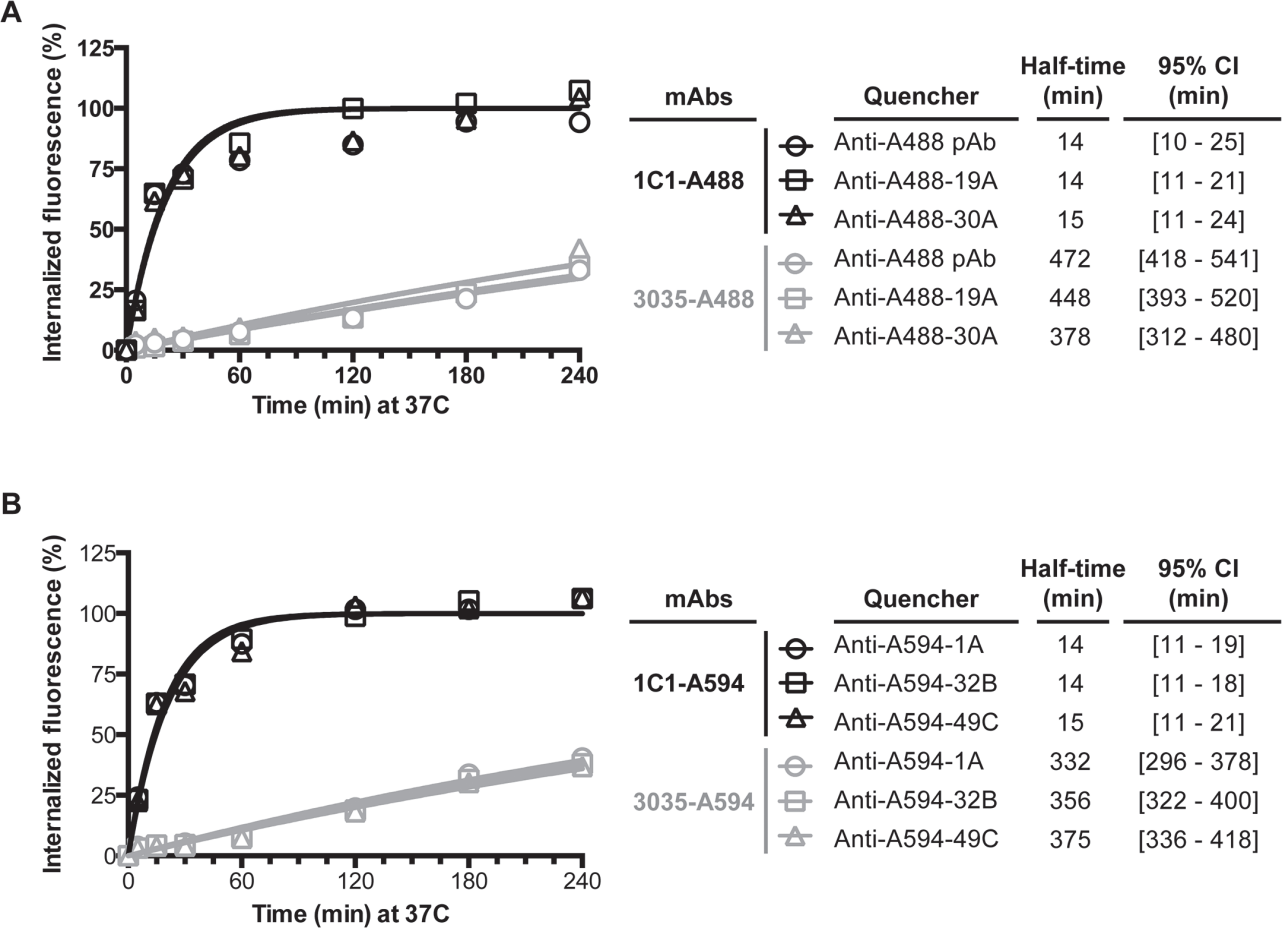
Internalization measurements of the anti-EphA2 mAbs with the anti-Alexa Fluor mAbs. Cellular uptake of 1C1-A488 or 3035-A488 (**A**) and 1C1-A594 or 3035-A594 (**B**) over a 4-hr time course. Upon labeling with the antibody conjugate, PC-3 cells were pulsed at 37°C for up to 4-hr. Surface fluorescence was subsequently quenched with 1 of 2 anti-A488 mAbs (**A**) or 1 of 3 anti-A594 mAbs (**B**). A rabbit anti-A488 polyclonal was used as a benchmark for the A488 conjugated mAbs. Percent internalization was calculated as described in the Material and Methods section. One representative experiment of multiple is shown. An independent experiment for the anti-A488 antibodies is shown in S1 Fig.

Internalization kinetics of surface-bound 1C1-A488, 1C1-A594, 3035-A488 and 3035-A594 were measured on PC-3 cells. For 1C1-A488 and 1C1-A594 internalization rates were similar, with fast uptake of at least 50% of the antibody conjugates in the first 15 min and slower uptake of at least another 45% of the conjugates in the subsequent 165 min (Fig 4). Using a one-phase association model, the times at which internalized fluorescence was equal to fifty percent (half-times) for the 1C1 conjugates were 14 to 15 min. Conversely, both 3035-A488 and 3035-A594 showed slow internalization kinetics: in the first 60 min of the assay less than 10% of either 3035 conjugate was internalized, and at the 3-hr time point only 25% was internalized (Fig 4). In comparison to 1C1’s half-times, 3035’s half-times were at least 22-fold greater, between 332 and 472 min (Fig 4). The 4-hr time course did not capture the 3035 half-times, resulting in relatively wide 95% confidence intervals for 3035 (Fig 4).

When conducting the internalization assay for either the 1C1-A488 or 3035-A488 conjugate, the anti-A488 mAbs 19A and 30A and the polyclonal benchmark generated similar uptake rates for each antibody conjugate (Fig 4A). In an independent experiment, the three anti-A594 mAbs also produced equivalent uptake curves for each antibody-A594 conjugate (Fig 4B).

To establish whether the amount of dye conjugated to the antibody affected its internalization, we compared two 1C1-A488 conjugates with a three-fold difference in dye load (S1 Fig). Despite the difference in dye load, the half-times for 1C1-A488 and 1C1-A488_v2 were equivalent (S1 Fig).

In summary, internalization measurements for the rapidly internalizing mAb 1C1 and the slowly internalizing mAb 3035 were equivalent across different Alexa fluorophores, dye-loads and anti-Alexa Fluor mAbs. To corroborate the difference in internalization between the two anti-EphA2 mAbs, immunofluorescence experiments were performed.

### Validation of flow cytometry–based internalization assay with immunofluorescence microscopy

**Fig 5:**
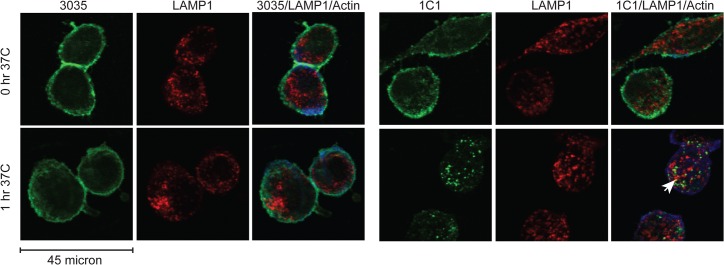
Surface-bound 1C1 is internalized, while 3035 shows little cellular uptake. Surface-bound 3035 or 1C1 was internalized in PC-3 cells for 0 or 1 hr at 37°C and processed for confocal immunofluorescence microscopy with the lysosomal marker LAMP1 (red color) and the actin cytoskeleton marker Phalloidin (blue color). Scale bar, 45 micron. White arrows illustrate colocalization. Representative staining from one of multiple independent experiments is shown.

While a large portion of 1C1 internalized after 1 hr, little to no 3035 appeared to be internalized (Fig 5). Some internalized 1C1 localized to the lysosome based on the lysosomal marker LAMP1 (Fig 5), a marker commonly used to assess antibody and ADC internalization [7, 11–13]. Both at the 0-hr and 1-hr time points, 3035 was predominantly found on the cell surface (Fig 5). Thus, microscopy-based assessment of mAb internalization was consistent with the flow-based internalization assay. Importantly, the flow-based internalization assay allowed a more rapid assessment of internalization compared to immunofluorescence microscopy.

### Anti-Alexa Fluor mAbs in dual-label assays

**Fig 6:**
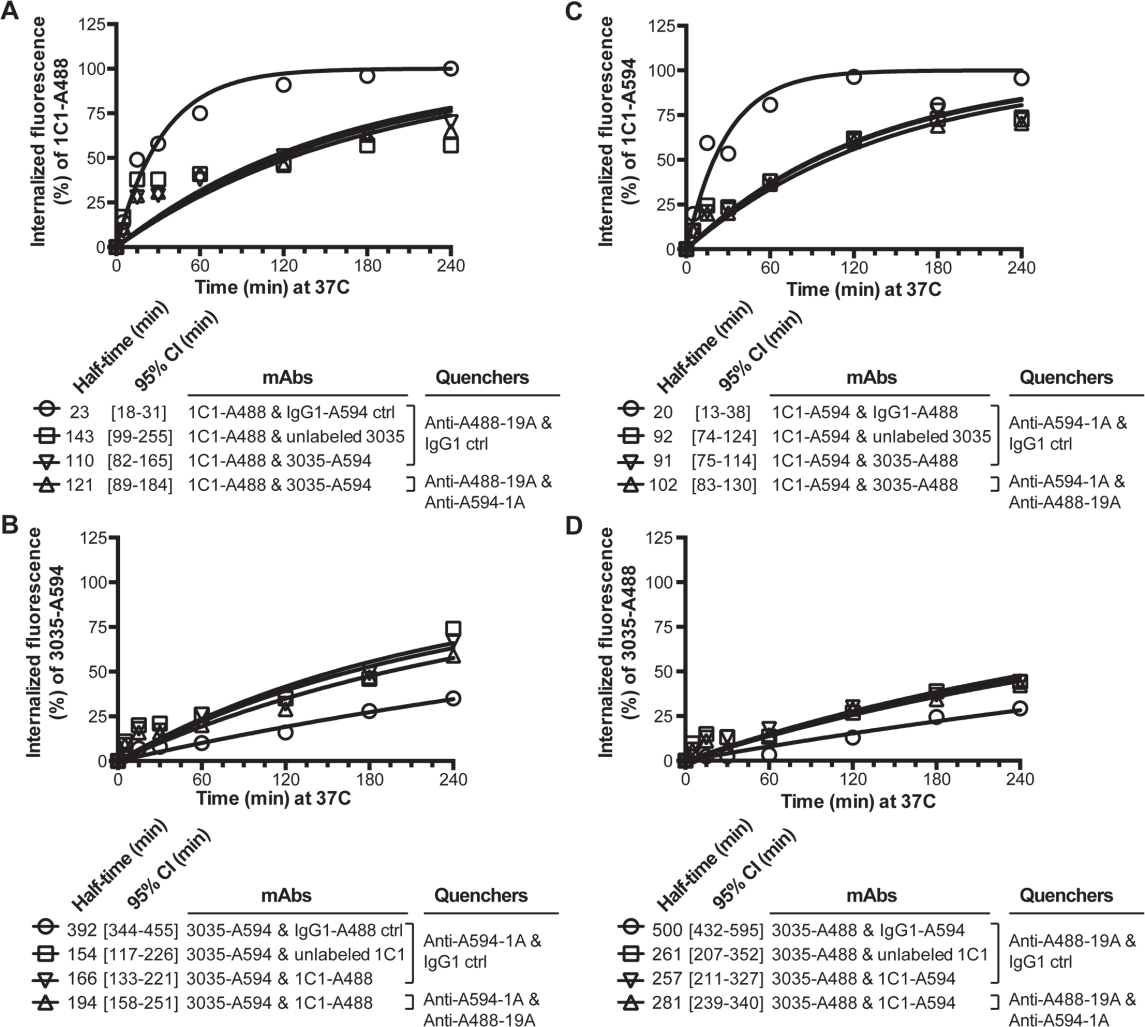
Internalization interplay between two anti-EphA2 mAbs measured with anti-Alexa Fluor mAbs. (**A**) Cellular uptake of 1C1-A488 in the presence of an IgG1-A594 control, unlabeled 3035 or 3035-A594 over a 4-hr time course. Surface A488 fluorescence was quenched with anti-A488-19A in the presence of the chimeric IgG1 isotype control or anti-A594-1A. (**B**) Cellular uptake of 3035-A594 in the presence of an IgG1-A488 control, unlabeled 1C1 or 1C1-A488 over a 4-hr time course. Surface A594 fluorescence was quenched with anti-A594-1A in the presence of the chimeric IgG1 isotype control or anti-A488-19A. The sample incubated with 1C1-A488 and 3035-A594 and quenched with anti-A488-19A and anti-A594-1A is analyzed for A488 fluorescence in **A** and A594 fluorescence in **B**. One representative experiment of multiple is shown. (**C, D**) Independent experiment in which the layout is reciprocal to the layout shown in **A** & **B**: 1C1 is labeled with A594 (**C**) instead of A488 (**A**), and the anti-Alexa Fluor mAbs and controls are changed accordingly. 3035 is labeled with A488 (**D**) instead of A594 (**A**), and the anti-Alexa Fluor mAbs and controls are changed accordingly. The sample incubated with 1C1-A594 and 3035-A488 and quenched with anti-A594-1A and anti-A488-19A is analyzed for A594 fluorescence in **C** and A488 fluorescence in **D**. One representative experiment of multiple is shown.

When targeting a surface antigen with two mAbs, it is important to understand interplay between the two mAbs and their target [21–23]. With the two different anti-Alexa Fluor mAbs in place, we assessed interplay between the slowly internalizing mAb 3035 and the rapidly internalizing mAb 1C1. First, PC-3 cells were labeled with 1C1-A488 and 3035-A594. After incubating cells with mAbs for up to 4 hr, surface fluorescence was quenched with anti-A488-19A and anti-A594-1A. Relative to the control in which PC-3 cells were stained with 1C1-A488 and the chimeric IgG1 isotype control labeled with A594, 1C1-A488’s half-time increased from 23 to 116 min by co-incubation with 3035-A594 (Fig 6A, circle versus triangle, respectively). A second control in which 3035-A594 was replaced with unlabeled 3035 showed a similar reduction in 1C1-A488’s internalization with a half-time of 124 min, demonstrating that dye conjugation to 3035 did not affect its activity (Fig 6A, square). The half-time of the third control sample in which cells were co-incubated with 1C1-A488 and 3035-A594 and only A488 fluorescence was quenched was 110 min, equivalent to the 121 min half-time of the sample in which cells were co-incubated with 1C1-A488 and 3035-A594 and both A488 and A594 fluorescence were quenched (Fig 6A, inverted triangle versus triangle, respectively), confirming that anti-A594-1A does not affect A488 fluorescence (S4 Fig). On the other hand, cellular uptake of 3035-A594 was increased by co-incubation with conjugated or unconjugated 1C1 (Fig 6B). 3035’s half-time decreased from 392 min to a half-time between 154 and 194 min by addition of naked or dye-conjugated 1C1.

In the reciprocal experiment in which PC-3 cells were stained with 3035-A488 and 1C1-A594, 3035 inhibited 1C1’s internalization to the same extent as in the first co-incubation experiment (Fig 6A & 6C). Conversely, 1C1 increased 3035’s cellular uptake to a degree similar to that seen in the first experiment (Fig 6B & 6D).

To compare internalization of the two anti-EphA2 mAbs, we used the one-phase association model to obtain half-times for all the internalization curves (Figs 4 & 6). For the samples with co-incubation of both anti-EphA2 mAbs, the 15-min and to a lesser degree the 30-min time points fell above the curve-fit (Fig 6, square, inverted triangle & triangle), indicating that the one-phase association equation underrepresents the magnitude of internalization in the first 60 min at 37°C. However, the remainder of the 4-hr time course was well captured by the equation, with single digit residuals on both sides of the curve.

**Fig 7:**
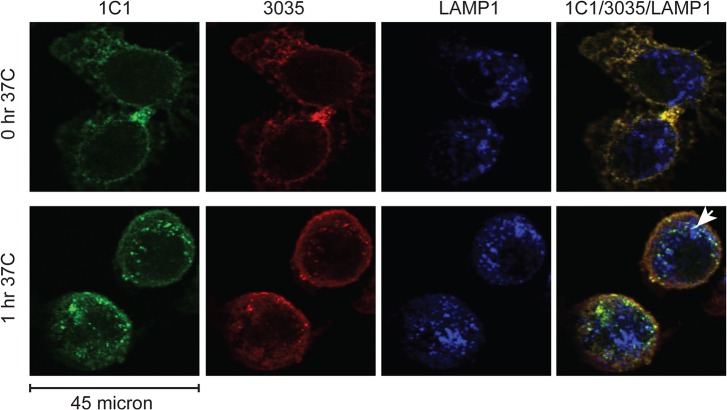
Internalization interplay between two anti-EphA2 mAbs monitored by immunofluorescence. Surface-bound 3035 (red color) and 1C1 (green color) were concurrently internalized in PC-3 cells for 0 or 1 hr at 37°C and processed for confocal immunofluorescence microscopy with the lysosomal marker LAMP1 (blue color). Scale bar, 45 micron. White arrows illustrate colocalization. Representative staining from one of multiple independent experiments is shown.

Confocal microscopy studies corroborated the validity of the flow-based studies. In PC-3 samples co-incubated with 1C1 and 3035, 1C1 increased the cellular uptake of 3035 at the 1-hr time point (Fig 7). Furthermore, some 1C1 appeared to remain cell surface localized at the 1-hr time point, indicating that 3035 attenuated 1C1’s internalization (Fig 7). In agreement with the non-competitive binding of 1C1 and 3035 to EphA2, 1C1 and 3035 appeared to both localize to the cell surface at the 0-hr time point and within the cell at the 1-hr time point (Fig 7). The two mAbs affected each other’s internalization activity, most likely because both mAbs can simultaneously bind different epitopes of EphA2 (Fig 3).

## Discussion

In the studies described here, novel anti-Alexa Fluor mAbs recognizing two structurally different Alexa Fluor dyes were developed and validated. We demonstrated that the specificity of the anti-Alexa Fluor mAbs enabled both single-label and dual-label internalization assays for antibodies against a cell surface antigen. The anti-Alexa Fluor mAbs should have broad applicability, not only to monitor antibody internalization, but also for other applications, including Alexa Fluor detection [24, 25], signal enhancement [26, 27], protein trafficking [28, 29], cellular morphology [30, 31], and cellular uptake of viral particles [32]. Other Alexa Fluor–based applications, such as detection of newly synthesized viral RNA molecules, may also benefit from these anti-Alexa Fluor reagents [33].

The anti-Alexa Fluor mAbs were found to be highly specific to their respective Alexa fluorophore in the context of different carrier proteins and antibodies. Anti-A488 and anti-A594 mAbs with substantial quenching activity were prioritized for in-depth characterization. Two of three anti-A488 and three of three anti-A594 purified mAbs showed more than 85% quenching activity against their respective fluorophores in titration experiments. We validated the anti-A488 mAbs using a benchmark anti-A488 polyclonal antibody, a reagent that has been used widely for various single-dye experiments [7, 24–32]. The anti-A488 polyclonal benchmark reached a similar degree of quenching, albeit with a higher IC50, likely because a subset of the polyclonal preparation bound KLH and only a subset had quenching activity. The lower IC50 of the mAbs compared to the polyclonal benchmark allows one to use less reagent and potentially reduces artifacts due to high antibody concentration. Another advantage of the anti-Alexa Fluor mAbs compared to polyclonal preparations is that they can be made recombinantly and may be cloned into alternate antibody scaffolds. The anti-Alexa Fluor mAbs were not benchmarked against anti-fluorescein mAbs, because pH-sensitive fluorescence makes fluorescein unsuitable for internalization studies [34]. Importantly, cellular uptake curves for a particular mAb were equivalent across different dyes, dye-loads and anti-Alexa Fluor mAbs.

Our attempts to raise anti-Alexa Fluor 647 and anti-Alexa Fluor 750 mAbs with substantial quenching activity against their respective fluorophores were unsuccessful. Future endeavors to obtain quenching mAbs against additional fluorescent dyes could enable further multiplexing.

For the development of antibody-drug conjugates, antibodies with favorable cellular uptake rates need to be identified. The flow cytometry–based assay presented here enables a rapid and quantitative assessment of antibody internalization. A 4-hr time course and data acquisition on a flow cytometer can be completed within a day, while immunofluorescence microscopy is more tedious.

Antibodies against EphA2 show different degrees of internalization [15]. Immunofluorescence microscopy experiments showed that the anti-EphA2 mAb 1C1 efficiently internalized EphA2 [15, 20], while the non-competing anti-EphA2 mAbs 3F2 and 1C9 induced only weak to no internalization [15]. Here, we corroborated that 1C1 efficiently internalized, and showed that the non-competing mAb 3035 poorly internalized. Unlike previous immunofluorescence studies, the flow cytometry–based internalization assay enabled rapid quantitation of internalization over time. The internalization curves were summarized with a half-time statistic derived with a one-phase association equation. A more quantitative assessment of internalization may facilitate prioritization of lead antibodies with favorable cellular uptake curves for development of antibody therapies, like ADCs.

Because the anti-Alexa mAbs are highly specific to their respective fluorophore, they can be used not only in single-label, but also in dual-label techniques. In the dual-label internalization assay with two differentially labeled anti-EphA2 mAbs, uptake of 1C1 was attenuated by 3035, while 3035’s uptake was increased by 1C1. The interplay between these two mAbs is likely driven by the fact that these mAbs have different epitopes and functional effects on EphA2. In therapeutic antibody development, assessing epitope diversity enables prioritization of diverse collections of antibodies for further studies such as internalization [35]. Additional studies are required to elucidate the mechanism of interplay between 1C1 and 3035. Different mechanisms of interplay between antibodies have been reported. In the context of two non-competing anti-HER2 mAbs, trastuzumab increased pertuzumab’s disruption of HER-2 dimerization with EGFR and HER-2 [22]. In another example, two non-competing mAbs against MET synergistically inhibit HGF binding to MET [23].

In summary, the novel and renewable anti-Alexa Fluor mAbs described herein enable quantitative multiplex measurements of internalization and should facilitate numerous other experiments, from protein trafficking to cellular remodeling.

## Supporting Information

S1 Fig Apparent affinities of anti-EphA2 mAbs and internalization of anti-EphA2 mAbs with different dye loads. (PDF)

S2 Fig Immunofluorescence for anti-EphA2 mAb in two channels. (PDF)

S3 Fig Specificity of anti-Alexa Fluor antibodies. (PDF)

S4 Fig Specific quenching activity of anti-Alexa Fluor mAbs. (PDF)

S1 Table Variable region sequences of C1.18.4 and anti-Alexa Fluor mAbs. (PDF)
