# Correction: Quantitative Assessment of Antibody Internalization with Novel Monoclonal Antibodies against Alexa Fluorophores

**DOI:** 10.1371/journal.pone.0128729

**Published:** 2015-05-15

**Authors:** The PLOS ONE Staff

There are a number of errors in the headings for Fig 1B, “Quenching by anti-Alexa Fluor mAbs.” “Beads coated with 1C1-A488” and “Beads stained with 1C1-A488” should be “Beads coated with 1C1-A594” and “Beads stained with 1C1-A594”. Please see the corrected Fig 1 here.

**Fig 1:**
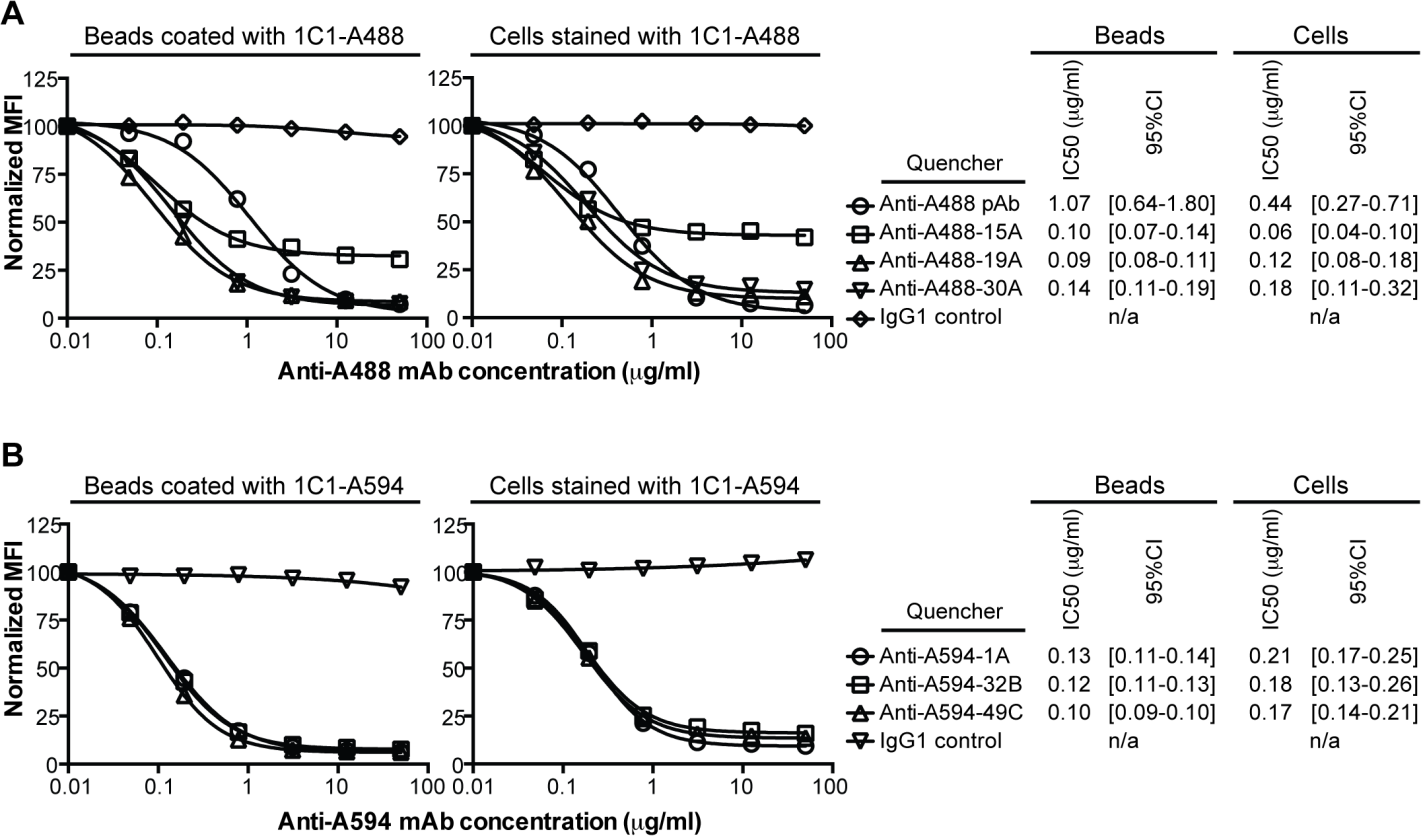
Quenching by anti-Alexa Fluor mAbs. (A) Fluorescence of Alexa Fluor 488 (A488) on microbeads coated with 1C1-A488 or PC-3 cells stained with 1C1-A488 was quenched with a titration of the benchmark, a rabbit anti-A488 polyclonal, or 1 of 3 anti-A488 mAbs. One representative experiment of multiple is shown. (B) Fluorescence of Alexa Fluor 594 (A594) on microbeads coated with 1C1-A594 or PC-3 cells stained with 1C1-A594 was quenched with a titration of 1 of 3 anti-A594 mAbs. One representative experiment of multiple is shown. (A, B) Median fluorescence intensities (MFIs) at each anti-A488 or anti-A594 mAb concentration were normalized against a buffer control. The chimeric IgG1 isotype control was used as a non-quenching mAb control. The IC50 values (microgram/ml) of quenching and the corresponding 95% confidence intervals (95% CI) are listed for both the microbead- and cell-based titrations.

There are errors in the equation in the second to last sentence of the penultimate paragraph of the subsection “Flow cytometry-based internalization assay using cell surface fluorescence quenching.” The correct sentence should be: Internalized fluorescence was calculated from quenched and non-quenched sample data by correcting for incomplete surface quenching: 1 – (N_1_ – Q_1_)/(N_1_ – (N_1_Q_0_/N_0_)) with N_1_ = Unquenched MFI at each time point (t_1_); Q_1_ = Quenched MFI at t_1_; Q_0_ = Quenched MFI for the sample kept on ice (t_0_); N_0_ = Unquenched MFI at t_0_. The publisher apologizes for the error.

There are errors in the equation in the first sentence of the last paragraph of the subsection “Flow cytometry-based internalization assay using cell surface fluorescence quenching.” The correct sentence should be: Internalized fluorescence was plotted and a curve fit was obtained in Prism by nonlinear regression with the one-phase association equation: Y = Y_0_ + (Plateau – Y_0_) * (1 – exp(-K*x)) with Y_0_ = 0 when X (time) is zero and Plateau = 100. The publisher apologizes for the error.
