# Correction: Quantitative Assessment of Antibody Internalization with Novel Monoclonal Antibodies against Alexa Fluorophores

**DOI:** 10.1371/journal.pone.0130106

**Published:** 2015-06-12

**Authors:** The PLOS ONE Staff

There is an error in the Correction published on May 15, 2015. The corrected headings for Fig 1B are “Cells stained with 1C1-A488” and “Cells stained with 1C1-A594”, not “Beads stained with 1C1-A488” and “Beads stained with 1C1-A594”. The publisher apologizes for the error. The correct text is:

There are a number of errors in the headings for Fig 1B, “Quenching by anti-Alexa Fluor mAbs.” “Beads coated with 1C1-A488” and “Cells stained with 1C1-A488” should be “Beads coated with 1C1-A594” and “Cells stained with 1C1-A594”. Please see the corrected Fig 1 here.

**Fig 1:**
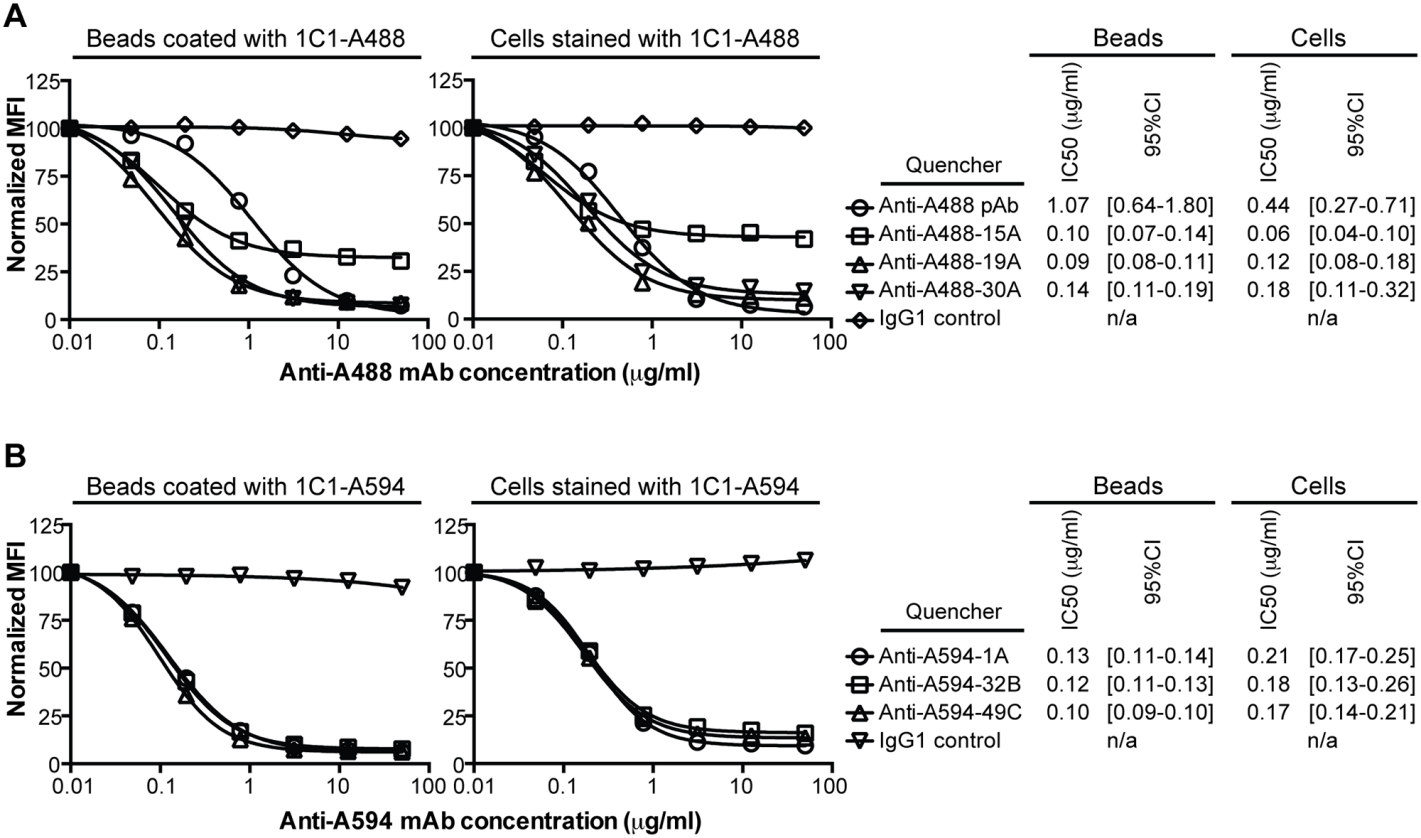
Quenching by anti-Alexa Fluor mAbs. (A) Fluorescence of Alexa Fluor 488 (A488) on microbeads coated with 1C1-A488 or PC-3 cells stained with 1C1-A488 was quenched with a titration of the benchmark, a rabbit anti-A488 polyclonal, or 1 of 3 anti-A488 mAbs. One representative experiment of multiple is shown. (B) Fluorescence of Alexa Fluor 594 (A594) on microbeads coated with 1C1-A594 or PC-3 cells stained with 1C1-A594 was quenched with a titration of 1 of 3 anti-A594 mAbs. One representative experiment of multiple is shown. (A, B) Median fluorescence intensities (MFIs) at each anti-A488 or anti-A594 mAb concentration were normalized against a buffer control. The chimeric IgG1 isotype control was used as a non-quenching mAb control. The IC50 values (microgram/ml) of quenching and the corresponding 95% confidence intervals (95% CI) are listed for both the microbead- and cell-based titrations.
