# Supplementary material for: Quantitative Assessment of Antibody Internalization with Novel Monoclonal Antibodies against Alexa Fluorophores
Source: PLoS One. 2015 Apr 20;10(4):e0124708. doi: 10.1371/journal.pone.0124708 (PMC4403856; doi:10.1371/journal.pone.0124708)
Supplement: S1 Fig — (PDF) [file pone.0124708.s001.pdf]

A

Antibody	K_D (nM)	k_{on} ($x10^5$ M^{-1} s^{-1})	k_{dis} ($x10^{-3}$ s^{-1})
1C1	110 ± 9	0.93 ± 0.05	10.3 ± 0.6
1C1-A488	96.9 ± 13.0	1.12 ± 0.09	10.8 ± 1.1
1C1-A488_v2	115 ± 42	1.39 ± 0.13	15.7 ± 4.4
1C1-A594	62.3 ± 26.7	1.75 ± 0.06	8.72 ± 1.04
3035	3.66 ± 0.16	3.33 ± 0.11	1.22 ± 0.04
3035-A488	3.40 ± 0.15	3.75 ± 0.21	1.27 ± 0.03
3035-A594	1.87 ± 0.36	4.79 ± 0.73	0.88 ± 0.06

C

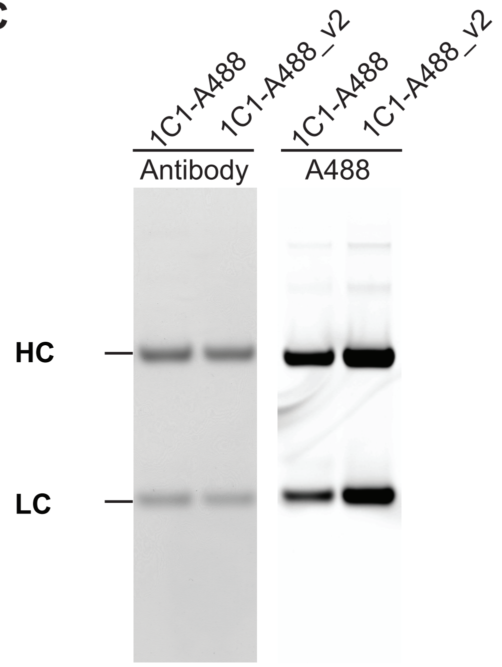

Relative intensities				
HC	132	129	711	2061
LC	87	83	372	913

B

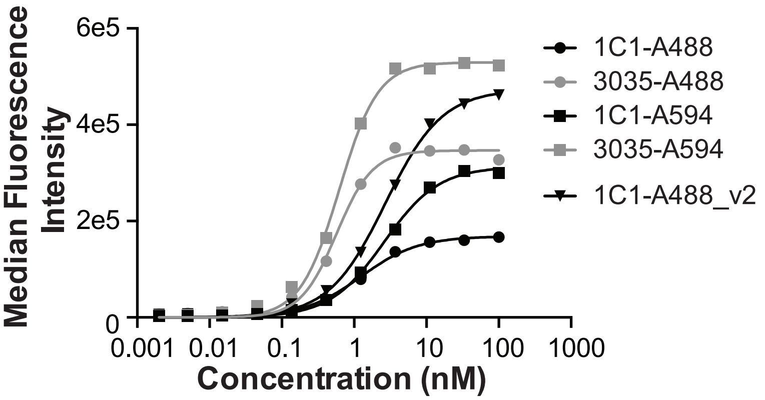

Antibody Conjugate	Kd [nM]	95% CI (nM)
1C1-A488	1.23	0.92 - 1.53
3035-A488	0.57	0.46 - 0.67
1C1-A594	2.60	2.25 - 2.95
3035-A594	0.62	0.55 - 0.70
1C1-A488_v2	2.70	2.34 - 3.06

D

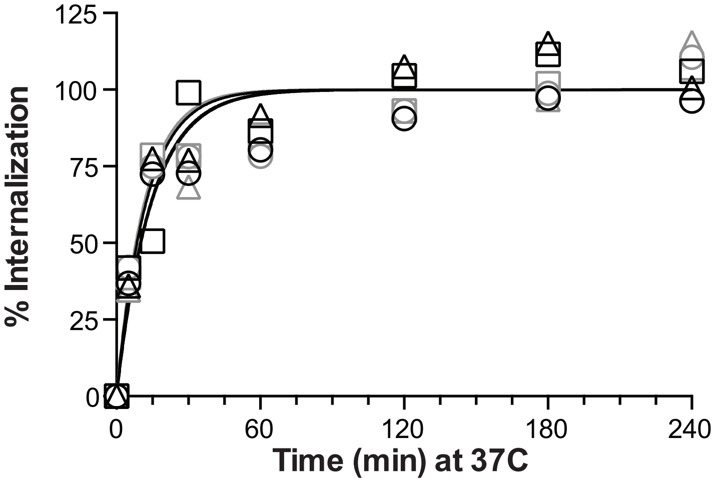

	Quencher	Half-time (min)	95% CI (min)
**1C1-A488**	Anti-Alexa488 pAb	10	[7 - 20]
	Anti-Alexa488-19A	10	[7 - 20]
	Anti-Alexa488-30A	9	[6 - 15]
**1C1-A488_v2**	Anti-Alexa488 pAb	8	[5 - 18]
	Anti-Alexa488-19A	8	[5 - 14]
	Anti-Alexa488-30A	10	[6 - 23]

S1 Fig. Apparent affinities of anti-EphA2 mAbs and internalization of anti-EphA2 mAbs with different dye loads

(**A**) Kinetic measurement values for naked and dye-conjugated mAbs. The standard deviation is derived from a triplicate data set. (**B**) Apparent Kd of 1C1 and 3035 conjugated to either A488 or A594. For 1C1, the apparent Kd for a second A488 conjugate, 1C1-A488_v2, is also shown. (**C**) Coomassie stain (antibody) and A488 dye load of two 1C1-A488 conjugates. While 1C1-A488 had relative intensities of 370 on the light chain and 910 on the heavy chain, the second batch of 1C1-A488 (1C1-A488_v2) had relative intensities of 710 on the light chain and 2060 on the heavy chain. (**D**) Percent internalizaition of two 1C1-A488 conjugates with a 3-fold difference in dye load between the two conjugates over a 4-hr time course.
