# Supplementary material for: Quantitative Assessment of Antibody Internalization with Novel Monoclonal Antibodies against Alexa Fluorophores
Source: PLoS One. 2015 Apr 20;10(4):e0124708. doi: 10.1371/journal.pone.0124708 (PMC4403856; doi:10.1371/journal.pone.0124708)
Supplement: S2 Fig — (PDF) [file pone.0124708.s002.pdf]

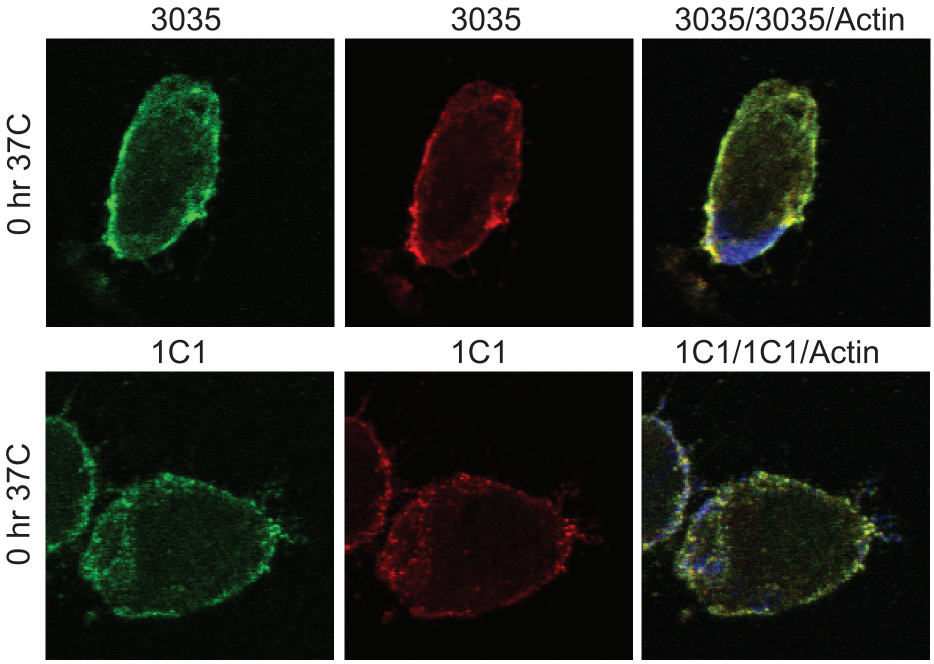

S2 Fig. Immunofluorescence for anti-EphA2 mAb in two channels.
Surface-bound 3035 or 1C1 on PC-3 cells was processed for immunofluorescence and detected in two channels (green and red) by confocal microscopy. The actin cytoskeleton was detected with Alexa Fluor 647 Phalloidin.
