# Supplementary material for: Quantitative Assessment of Antibody Internalization with Novel Monoclonal Antibodies against Alexa Fluorophores
Source: PLoS One. 2015 Apr 20;10(4):e0124708. doi: 10.1371/journal.pone.0124708 (PMC4403856; doi:10.1371/journal.pone.0124708)
Supplement: S3 Fig — (PDF) [file pone.0124708.s003.pdf]

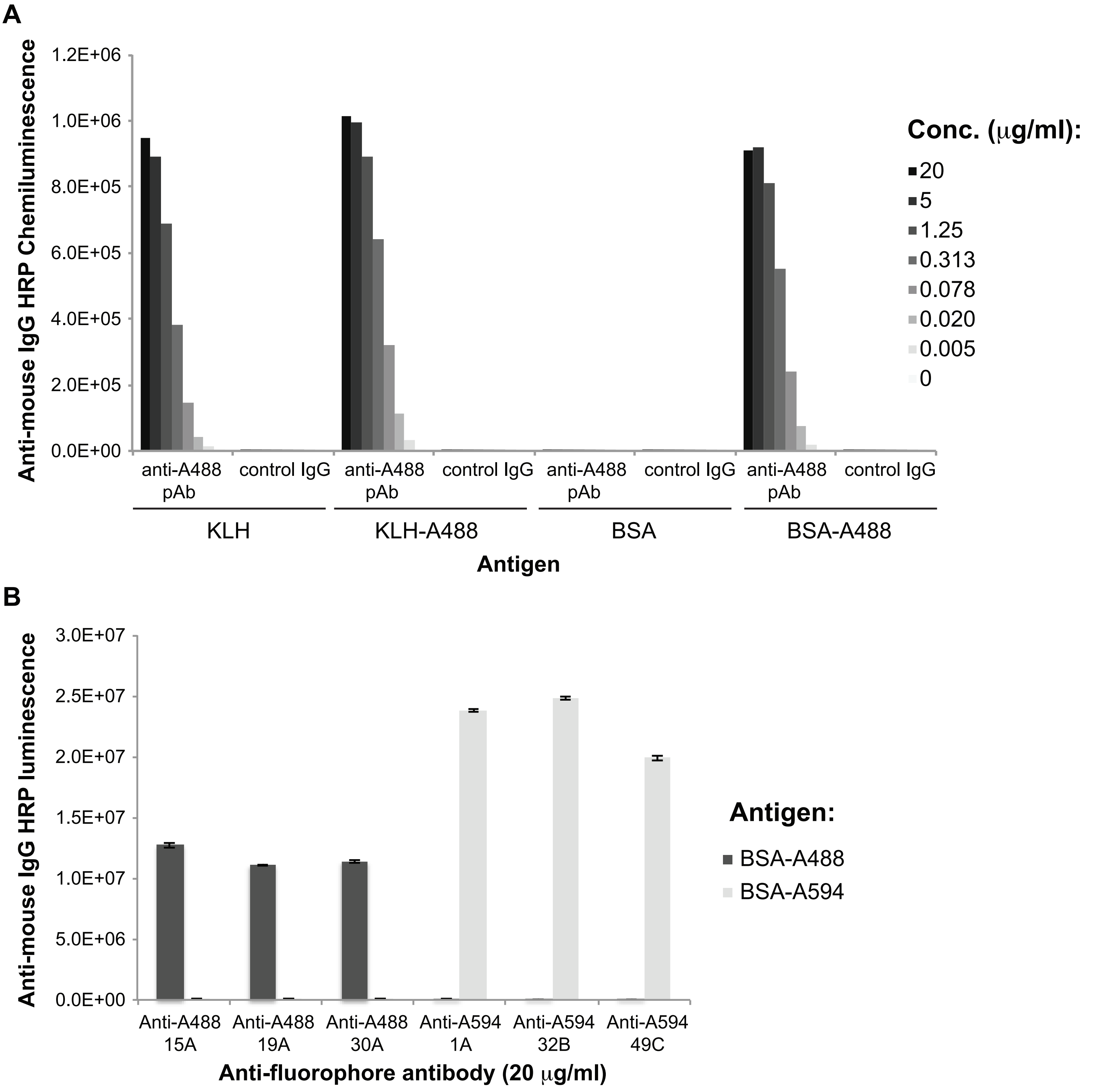

S3 Fig. Specificity of anti-Alexa Fluor antibodies.
(**A**) Luminescence-based binding of the quenching benchmark, a rabbit anti-A488 polyclonal antibody preparation, to keyhole limpet hemocyanin protein (KLH) and A488, coupled to either KLH or bovine serum albumin (BSA). A 7-point titration of the anti-A488 polyclonal starting at 20 μg/ml on KLH, KLH-A488, BSA and BSA-A488.
(**B**) The anti-A488 and anti-A594 mAbs do not cross-react with A594 and A488, respectively. Luminescence-based binding of the prioritized anti-A488 and anti-A594 antibody supes to BSA-A488 and BSA-A594.
