# Supplementary material for: Quantitative Assessment of Antibody Internalization with Novel Monoclonal Antibodies against Alexa Fluorophores
Source: PLoS One. 2015 Apr 20;10(4):e0124708. doi: 10.1371/journal.pone.0124708 (PMC4403856; doi:10.1371/journal.pone.0124708)
Supplement: S4 Fig — (PDF) [file pone.0124708.s004.pdf]

A

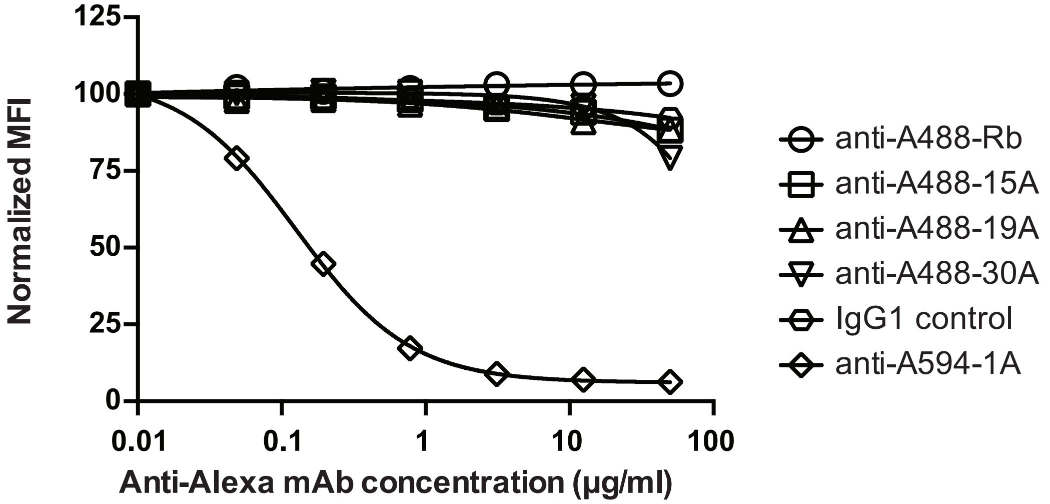


B

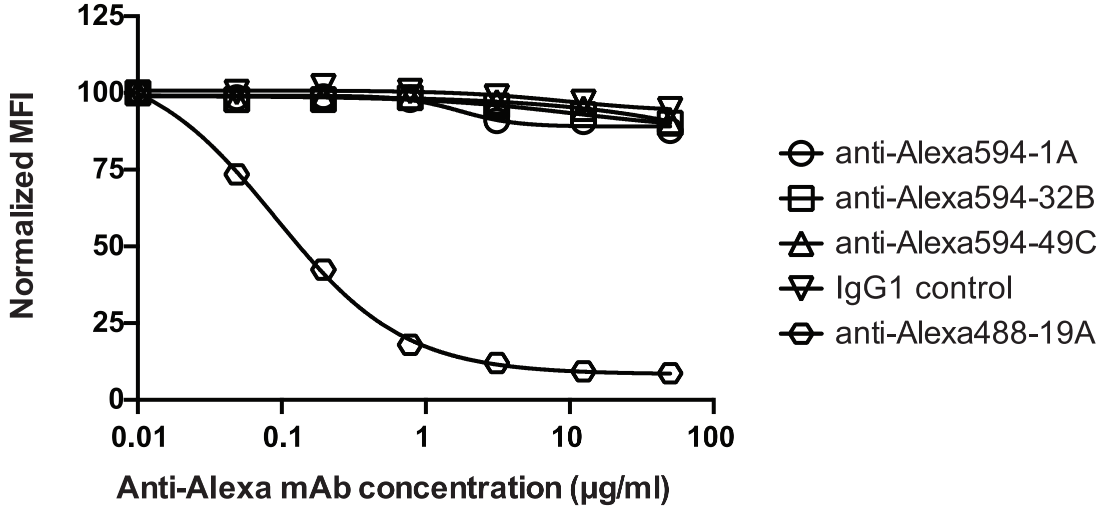


**S4 Fig. Specific quenching activity of anti-Alexa Fluor mAbs.**
(**A**) Fluorescence of A594 on microbeads coated with 1C1-A594 was quenched with a titration of the benchmark, a rabbit anti-A488 polyclonal, or 1 of 3 anti-A488 mAbs, or the positive control anti-A594-1A. (**B**) Fluorescence of A488 on microbeads coated with 1C1-A488 was quenched with a titration of 1 of 3 anti-A594 mAbs, or the positive control anti-A488-19A. (**A, B**) Median fluorescence intensities (MFIs) at each anti-A488 or anti-A594 mAb concentration were normalized against a buffer control. An IgG1 was used as a non-quenching mAb control.
