# Supplementary material for: Quantitative Assessment of Antibody Internalization with Novel Monoclonal Antibodies against Alexa Fluorophores
Source: PLoS One. 2015 Apr 20;10(4):e0124708. doi: 10.1371/journal.pone.0124708 (PMC4403856; doi:10.1371/journal.pone.0124708)
Supplement: S1 Table — (PDF) [file pone.0124708.s005.pdf]

S1 Table. Variable region sequences of C1.18.4 and anti-Alexa Fluor mAbs.

Heavy chain sequence (HC) and light chain (LC) nucleotide sequences are shown.

The mouse IgG2a; kappa light chain control C1.18.4	
**C1.18.4 HC**	ATGAGATGGAGCTGTATCATCCTCTTCTTGGTAGCAACAGCTACAGGTGTCAATTCCCAGGTCCAACTGCAGCAGCCTGGAGCT<br>GAGCTTGTGATGCCTGGGGCTTCAGTGAAGATGTCCTGCAAGGCTTCTGGCTACACCTTCACTGACTACTGGATGCACTGGGTG<br>AAGCAGAGGCCTGGACAAGGCCTTGAGTGGATCGGAGAGATTGATATTTCTGATAGTTATACTAACTACAATCAAAAGTTCAAGG<br>GCCAGGCCACATTGACTGTAGACGAATCCTCCAACACAGCCTACATGCAGCTCAGCAGCCTGACATCTGAGGACTCTGCGGTCT<br>ATTTCTGTGCAAGAGGGAGAAGGGGGTACTTCGATGTCTGGGGCGCAGGGACCACGGTCACCGTCTCCTCA
**C1.18.4 LC**	ATGGATTTTCAAGTGCAGATTTTCAGCTTCCTGCTAATGAGTGCCTCAGTCATAATGTCCAGGGGACAAATTGTTCTCACCCAGT<br>CTCCAGCACTCATGTCTGCATCTCTAGGGGAGAAGGTCACCATGACCTGCAGTGCCAGCTCAAGTATAAATTACATGTATTGGTA<br>CCAACAGAAGCCAGGATCCTCCCCCAAACCCTGGATTTATCTCACATCCAACCTGGCTTCTGGAGTCCCTGCTCGCTTCAGTGG<br>CAGTGGGTCTGGGACCTCTTATTCTCTCACAATCAGCAGCATGGAGGCTGAAGATGCTGCCACTTATTACTGTCAGCAGTGGAG<br>TAGTAATCCCCTCACGTTCGGTGCTGGGACCAAGCTGGAGCTGAAA

Anti-A488 mAbs	
**15A HC**	ATGGGCAGGCTTACTTCTTCATTCCTGCTACTGATTGTCCCTGCATATGTCCTGTCCCAGGTTACTCTGAAAGAGTCTGGCCCTG<br>GGATATTGCAGCCCTCCCAGACCCTCAGTCTGACTTGTTCTTTCTCTGGGTTTTCACTGAGCACTTCTGGTATGGGTGTAGGCTG<br>GATTCGTCAGCCATCAGGGAAGGGTCTGGAGTGGCTGGCACACATTTGGTGGGATGATGTCAAGCGCTATAACCCAGCCCTGA<br>AGAGCCGACTGACTATCTCCAAGGATACCTCCAGGAGCCAGGTATTCCTCAAGATCGCCAGTGTGGACACTGCAGATACTGCCA<br>CATACTACTGTGTTCGAATTGATTACTACGGTAGTAGCTACGCTTACTGGGGCCAAGGGACTCTGGTCACTGTCTCTGCA
**15A LC**	ATGGATTCACAGGCCCAGGTTCTTATATTGCTGCTGCTATGGGTATCTGGTACCTGTGGGGACATTGTGATGTCACAGTCTCCAT<br>CCTCCCTGGCTGTGTCAGCAGGAGAGAAGGTCACTATGAACTGCAAATCCAGTCAGAGTCTGCTCAACAGTAGAACCCGAAAGA<br>ACTACTTGGCTTGGTACCAGCAGAAACCAGGGCAGTCTCCTAAACTGCTGATCTACTGGGCATCCACTAGGGAATCTGGGGTCC<br>CTGATCGCTTCACAGGCAGTGGATCTGGGACAGATTTCACTCTCACCATCAGCAGTGTGCAGGCTGAAGACCTGGCAGTTTATT<br>ACTGCAAGCAATCTTATAATCTTTACACGTTCGGAGGGGGGACCAAGCTGGAAATAAAA
**19A HC**	ATGGGTTGGCTGTGGAACTTGCTATTCCTGATGGCAGCTGCCCAAAGTGCCCAAGCACAGATCCAGTTGGTACAGTCTGGACCT<br>GAGCTGAAGAAGCCTGGAGAGACAGTCAAGATCTCCTGCAAGGCTTCTGGGTATACCTTCACAACCTATGGAATGACCTGGGTG<br>AAACAGGCTCCAGGAAAGGGTTTAAAGTGGATGGGCTGGATAAACACCTACTCTGGAGTGCCAACATATGCTGATGACTTCAAG<br>GGACGGTTTGCCTTCTCTTTGGAAACCTCTGCCAGCACTGCCTATTTGCAGATCAACAACCTCAAAAATGAGGACACGTCTACAT<br>ATTTCTGTGCAAGAAGAAGAAGGAGGGAAATTTATTACTACGGTAGTACCTACGGGTGGTACTTCGATGTCTGGGGCACAGGGA<br>CCACGGTCACCGTCTCCTCA
**19A LC**	ATGGACATGATGGTCCTTGCTCAGTTTCTTGCATTCTTGTTGCTTTGGTTTCCAGGTGCAAGATGTGACATCCTGATGACCCAAT<br>CTCCATCCTCCATGTCTGGATCTCTGGGAGACACAGTCAGCATCACTTGCCATGCAAGTCAGGGCATTAGCAGTAATATAGGGT<br>GGTTGCAGCAGAAACCAGGGAAATCATTTAAGGGCCTGATCTATCATGGAACCAACTTGGAAGATGGAGTTCCATCAAGGTTCA<br>GTGGCAGTGGATCTGGAACAGATTATTCTCTCACCATCAGCAGCCTGGAATCTGAAGATTTTGCAGACTATTACTGTGTACAGTA<br>TGCTCAGTTTCCGTACACGTTCGGAGGGGGGACCAAGCTGGAAATAAAA
**30A HC**	ATGGGATGGAGCTCTATCATCCTCTTCTTGGTAGCAACAGCTACAGGTGTCCACTCCCAGGTCCAACTGCAGCAGCCTGGGGCT<br>GAGCTGGTGAGGCCTGGAGCTTCAGTGAAGCTGTCCTGCAAGGCTTCTGGCTACTCCTTCACCAGCTACTGGATGAACTGGGT<br>GAAGCAGAGGCCTGGACAAGGCCTTGAGTGGATTGGCATGATTCATCCTTCCGATAGTGAAACTAGGTTAAATCAGAAGTTCAA<br>GGACAAGGCCACATTGACTGTAGACAAATCCTCCAGCACAGCCTACATGCAACTCAGCAGCCCGACATCTGAGGACTCTGCGGT<br>CTATTACTGTGCAAGAGGGAGGGGCGTTTACTACGCTGGTAGCCCCTTCTACTTTGACTACTGGGGCCAAGGCACCACTCTCAC<br>AGTCTCCTCA
**30A LC**	ATGGAGAAAGACACACTCCTGCTATGGGTCCTGCTTCTCTGGGTTCCAGGTTCCACAGGTGACATTGTACTGACCCAATCTCCA<br>GCTTCTTTGGCTGTGTCTCTAGGGCAGAGGGCCACCATCTCCTGCAGAGCCAGCGAAAGTGTTGATAATTATGGCATTAGTTTTA<br>TGAACTGGTTCCAACAGAAACCAGGACAGCCACCCAAACTCCTCATCTATGCTGCATCCAACCAAGGATCCGGGGTCCCTGCCA<br>GGTTTAGTGGCAGTGGGTCTGGGACAGACTTCAGCCTCAACATCCATCCTATGGAGGAGGATGATACTGCAATGTATTTCTGTC<br>AGCAAAGTAAGGAGGTTCCGTGGACGTTCGGTGGAGGCACCAAGCTGGAAATCAAA

Anti-A594 mAbs	
1A HC	ATGTACTTGGGACTGAACTGTGTATTCATAGTTTTTCTCTTAAAAGGTGTCCAGAGTGAAGTGAAGCTTGAGGAGTCTGGAGGAG<br>GCTTGGTGCAACCTGGAGGATCCATGAAACTCTCTTGTGCTGCCTCTGGATTCACTTTTAGTGACGCCTGGATGGACTGGGTCC<br>GCCAGTCTCCAGAGAAGGGGCTTGAGTGGGTTGCTGAAATTAGAAACAAAGCTAATAATCATGCAACATACTATGCTGAGTCTGT<br>GAAAGGGAGGTTCACCATCTCAAGAGATGATTCCAAAAGTAGTGTCTACCTGCAAATGAATAGCTTAAGAGCTGAAGACACTGG<br>CATTTATTACTGTACGTCGGTCTGGCTTGCTTACTGGGGCCAAGGGACTCTGGTCACTGTCTCTGCA
1A LC	ATGAAGTTGCCTGTTAGGCTGTTGGTGCTGATGTTCTGGATTCCTGCTTCCAGCAGTGATGTTGTGATGACCCAAACTCCACTCT<br>CCCTGCCTGTCAGTCTTGGAGATCAAGCCTCCATCTCTTGCAGATCTAGTCAGAGCCTTGTACACAGTAATGGAAACACCTATTT<br>ACATTGGTACCTGCAGAAGCCAGGCCAGTCTCCAAAGCTCCTGATCTACAAAGTTTCCAACCGATTTTCTGGGGTCCCAGACAG<br>GTTCAGTGGCAGTGGATCAGGGACAGATTTCACACTCAAGATCAGCAGAGTGGAGGCTGAGGATCTGGGAGTTTATTTCTGCTC<br>TCAAAGTACACATGTTCCGCTCACGTTCGGTGCTGGGACCAAGCTGGAGCTGAAA
32B HC	ATGGGATGGAGCTGGATCTTTCTCCTCTTCCTGTCAGGAACTGCAGGTGTCCTCTCTGAGGTCCAGCTGCAACAGTCTGGACCT<br>GAGCTGGTGAAGCCTGGGGCTTCAGTGAAGATACCCTGCAAGGCTTCTGGATACACATTCACTGACTACAACATGGACTGGGTG<br>AAGCAGAGCCATGGAAAGAGCCTTGAGTGGATTGGAGATATTAATCCTAACAATGGTGGTCCTATCTACAACCAGAAGTTCAAG<br>GGCAAGGCCACATTGACTATAGACAAGTCCTCCAGCACAGCCTACATGGAGCTCCGCAGCCTGACATCTGAGGACACTGCAGT<br>CTATTACTGTGCAGGCCGGTGGTTTGCTTACTGGGGCCAAGGGACTCTGGTCACTGTCTCTGCA
32B LC	ATGAAGTTGCCTGTTAGGCTGTTGGTGCTGATGTTCTGGATTCCTGCTTCCAGCAGTGATGTTGTGATGACCCAAACTCCACTCT<br>CCCTGCCTGTCAGTCTTGGAGATCAAGCCTCCATCTCTTGCAGATCTAGTCAGAGCCTTGTACACAGTAATGGAAACACCTATTT<br>ACATTGGTACCTGCAGAAGCCAGGCCAGTCTCCAAAGCTCCTGATCTACAAAGTTTCCAACCGATTTTCTGGGGTCCCAGACAG<br>GTTCAGTGGCAGTGGATCAGGGACAGATTTCACACTCAAGATCAGCAGAGTGGAGGCTGAGGATCTGGGAGTTTATTTCTGCTC<br>TCAAAGTACACATGTTCCGTACACGTTCGGAGGGGGGACCAAGCTGGAAATAAAA
49C HC	ATGGGATGGAGCTCTATCATCCTCTTCTTGGTAGCAACAGCCTCAGGTGTCCACTCCCAGGTCCAACTGCAGCAACCTGGGTCT<br>GAGCTGGTGAGGCCTGGAGCTTCAGTGAAGCTGTCCTGCAAGGCTTCTGGCTACACATTCACCAGCTACTGGATGGACTGGGT<br>GAAGCAGAGGCCTGGACAAGGCCTTGAGTGGATTGGAAATATTTATCCTGGTAGTGGTAGTACTAACTACGATGAGAAGTTCAA<br>GAGCAAGGCCACACTGACTGTAGACACATCCTCCAGCACAGCCTACATGCAGCTCAGCAGCCTGACATCTGAGGACTCTGCGG<br>TCTATTACTGTACCTGTACCTGGTTTGCTTACTGGGGCCAAGGGACTCTGGTCACTGTCTCTGCA
49C LC	ATGAAGTTGCCTGTTAGGCTGTTGGTGCTGATGTTCTGGATTCCTGCCTCCAGCAGTGATGTTGTGATGACCCAAACTCCACTCT<br>CCCTGCCTGTCAGTCTTGGAGATCAAGCCTCCATCTCTTGCAGATCTAGTCAGAGCCTTGTACACAGTAATGGAAACACCTATTT<br>ACATTGGTACCTGCAGAAGCCAGGCCAGTCTCCAAAGCTCCTGATCTACAAAGTTTCCAACCGATTTTCTGGGGTCCCAGACAG<br>GTTCAGTGGCAGTGGATCAGGGACAGATTTCACACTCAAGATCAGCAGAGTGGAGGCTGAGGATCTGGGAGTTTATTTCTGCTC<br>TCAAAGTACACATGTTCCGTACACGTTCGGAGGGGGGACCAAGCTGGAAATAAAA
